# Association of anti-apoptotic Mcl-1L isoform expression with radioresistance of oral squamous carcinoma cells

**DOI:** 10.1186/1748-717X-7-135

**Published:** 2012-08-08

**Authors:** Vinayak C Palve, Tanuja R Teni

**Affiliations:** 1 Teni Lab, Advanced Centre for Treatment, Research and Education in Cancer (ACTREC), Tata Memorial Centre, Kharghar, Navi Mumbai, 410210, India

## Abstract

**Background:**

Oral cancer is a common cancer and a major health problem in the Indian subcontinent. At our laboratory Mcl-1, an anti-apoptotic member of the Bcl-2 family has been demonstrated to be overexpressed in oral cancers and to predict outcome in oral cancer patients treated with definitive radiotherapy. To study the role of Mcl-1 isoforms in radiation response of oral squamous carcinoma cells (OSCC), we investigated in the present study, the association of Mcl-1 isoform expression with radiosensitivity of OSCC, using siRNA strategy.

**Methods:**

The time course expression of Mcl-1 splice variants (Mcl-1L, Mcl-1S & Mcl-1ES) was studied by RT-PCR, western blotting & immunofluorescence, post-irradiation in oral cell lines [immortalized FBM (radiosensitive) and tongue cancer AW8507 & AW13516 (radioresistant)]of relatively differing radiosensitivities. The effect of Mcl-1L knockdown alone or in combination with ionizing radiation (IR) on cell proliferation, apoptosis & clonogenic survival, was investigated in AW8507 & AW13516 cells. Further the expression of Mcl-1L protein was assessed in radioresistant sublines generated by fractionated ionizing radiation (FIR).

**Results:**

Three to six fold higher expression of anti-apoptotic Mcl-1L versus pro-apoptotic Mcl-1S was observed at mRNA & protein levels in all cell lines, post-irradiation. Sustained high levels of Mcl-1L, downregulation of pro-apoptotic Bax & Bak and a significant (*P* < 0.05) reduction in apoptosis was observed in the more radioresistant AW8507, AW13516 versus FBM cells, post-IR. The ratios of anti to pro-apoptotic proteins were high in AW8507 as compared to FBM. Treatment with Mcl-1L siRNA alone or in combination with IR significantly (*P* < 0.01) increased apoptosis viz. 17.3% (IR), 25.3% (siRNA) and 46.3% (IR plus siRNA) and upregulated pro-apoptotic Bax levels in AW8507 cells. Combination of siRNA & IR treatment significantly (*P* < 0.05) reduced cell proliferation and clonogenic survival of radioresistant AW8507 & AW13516 cells, suggesting a synergistic effect of the Mcl-1L siRNA with IR on radiosensitivity. Interestingly, during the development of radioresistant sublines using FIR, high expression of Mcl-1L was observed.

**Conclusion:**

Our studies suggest that Mcl-1L isoform has an important role in the survival and radioresistance of OSCC and may be a promising therapeutic target in oral cancers.

## Introduction

Squamous cell carcinoma of the oral cavity (OSCC) is the most prevalent cancer in males of the Indian subcontinent and is predominantly associated with the tobacco-chewing habit [1]. Radiotherapy is an important treatment modality in oral cancer aiding in tumor size reduction and preservation of oral function [2]. Despite advances in radiotherapy techniques, OSCC patients frequently develop loco-regional recurrence resulting in 5-year survival rates that have remained unchanged for past few decades [3]. Hence for successful radiotherapy, it is crucial to understand the mechanisms involved in the development of radiation resistance in tumor cells.

Anti-apoptotic members of the Bcl-2 family are the key regulators of cellular apoptosis and their over expression has been shown to be associated with radio-resistance [4,5]. Mcl-1 (Myeloid cell leukemia-1), an anti-apoptotic member of the Bcl-2 gene family, is essential for development, differentiation, and proliferation [6]. The overexpression of Mcl-1 has also been reported in a variety of hematopoietic, lymphoid and solid tumors [7-9]. Our earlier studies demonstrate the overexpression of anti-apoptotic Mcl-1 transcripts & protein in oral tumors and cell lines [10]. Further, we have also demonstrated Mcl-1 to be a prognostic factor in oral cancer patients treated with definitive radiotherapy [11]. Earlier, Mcl-1 has been shown to contribute in resistance of cancer cells to chemotherapeutic agents, however reports on its role in radiation induced apoptosis and radioresistance are rare [12,13]. Further, the situation is complex due to the existence of distinct Mcl-1 isoforms having contrasting functions (anti-apoptotic Mcl-1L, pro-apoptotic Mcl-1S & Mcl-1ES)[14]. Earlier studies from our lab have demonstrated a five to ten fold higher expression of anti-apoptotic Mcl-1L transcript, versus the pro-apoptotic Mcl-1S in oral tumors [10]. Therefore, in the present study we wanted to investigate the association of Mcl-1 isoforms with radioresistance of oral cancer cells using siRNA strategy. To the best of our knowledge, no reports are available on the role of Mcl-1 splice variants in radiation response of OSCC.

The present study was undertaken to compare the time course profile of Mcl-1 splice variants and other Bcl-2 family members, post-radiation (IR) treatment in oral cell lines of differing radiosensitivities. Further, the effect of Mcl-1L knockdown alone or in combination with IR on cell proliferation, apoptosis and radiosensitivity of oral cells was investigated.

## Materials and methods

### Cell culture

Established AW8507 & AW13516 [10,15] & FBM (fetal buccal mucosa derived immortalized cell line) [16] were selected for the study due to their differing radiosensitivities. The cell lines were cultured in IMDM supplemented with 10% FBS (Gibco, US), 100 units/ml penicillin, 100 μg/ml streptomycin, 2mM L-glutamine and keratinocyte growth supplements only for FBM (Sigma, USA), in 5% CO_2_ at 37°C.

### Clonogenic Assay

Exponentially growing oral cells were harvested, counted and replated in duplicates. After 24 hrs, the cells were treated with different doses of IR (1, 2, 4, 6, 8, 10 Gy) using ^60^Co-γ radiator along with an untreated control. Cells were then incubated up to 14 days to form colonies which were fixed and stained with a mixture of glutaraldehyde (6.0% v/v) and crystal violet (0.5% w/v) and colonies (≥50 cells) were counted using a microscope. The percent plating efficiency and fraction surviving a given radiation dose were calculated based on the survival of non-irradiated cells as described earlier [17].

### Radiation Treatment

After 48 hrs of plating exponentially growing cells (6 × 10^3^ cells) were treated with IR (D_0_ dose) using ^60^Co-γ radiator as described earlier [13]. Cells were incubated upto different time points, harvested and stored in -80°C until use.

### RNA isolation

Cell pellets were placed in TRI reagent (Sigma, USA) and total cellular RNA was isolated according to the manufacturer’s protocol. The RNA was dissolved in DEPC-treated water and contaminating DNA was removed by DNaseI treatment (Sigma, USA). RNA integrity was analyzed by electrophoresis and samples were preserved at −80°C until analysis, as described earlier [10].

### Reverse transcriptase-polymerase chain reaction

cDNA was synthesized with 2 μg total RNA, using a First Strand cDNA synthesis kit (MBI Fermentas, Canada) according to the manufacturer’s instructions. The efficiency of cDNA synthesis and equal loading were assessed by ß-actin PCR. Mcl-1 isoforms (Mcl-1L, Mcl-1S & Mcl-1ES isoforms) were amplified by using primers (forward 5’ACGCGGTAATCGGACTCAACCT3’ and reverse 5’GCAGCACATTCCTGATGCCACCT3’), as reported earlier [10]. A separate PCR was performed to determine expression of Mcl-1ES isoform using forward (5’ACGCGGTAATCGGACTCAACCT3’) and reverse (5’GCAGCACATTCCTGATGCCACCT3’) primers as shown in Additional file 1. PCR products were resolved on 2% agarose gel containing ethidium bromide and quantitated using Gel-doc system (UVP, UK).

### Western blotting

Cell lysates were resolved on 12% SDS-PAGE gels and transferred onto PVDF membranes (Millipore, USA). Membranes were blocked with 5% skimmed milk in TBS for 2 hrs. Primary antibodies used were anti-Bax (1:150, Abcam, USA), anti-Mcl-1 (1:1000), anti-Bclxl (1:1000) and anti-ß-actin (1:2000) (Santa Cruz Biotechnology, USA). Secondary antibodies used were Horseradish peroxidase conjugated IgG (1:5000) (Santa Cruz Biotechnology, USA). Proteins were visualized with enhanced chemiluminescence kit (GE Healthcare, US). Densitometry analysis of developed X-ray film was performed using ImageJ software (NIH, Bethesda, MD). β-actin was used as loading control.

### Apoptosis detection by flow cytometry

The Annexin V-FITC apoptosis detection kit (Santa Cruz Biotechnology, CA) was used for the detection of apoptotic cells in the three oral cell lines, as per the manufacturer’s specifications. Briefly, cells were collected by trypsinization at different time points (Control, 1, 4, 24 & 48 hrs) post-IR treatment. Cells were washed; 2μg Annexin-V FITC & 10 μl PI were added, incubated in the dark for 15 min and analyzed on a flow cytometer (FACS Caliber, BD, USA).

### Immunofluorescence staining

Cells were grown on glass cover slips and Mcl-1 staining was performed at different time points in both FBM & AW8507, post-IR using an Alexa fluor-488 labeled secondary antibody (1:1000, Molecular Probes, USA), as described earlier [10]. The AW8507 cells were treated with siRNA (100nM) and/or exposed to IR as described above. The nuclear condensation and apoptosis was analyzed by DAPI (Sigma-Aldrich, USA) staining, cell counting and imaging was done by confocal microscope with LSM Image Browser 4.2 software (Carl Zeiss).

### Knockdown of Mcl-1L isoform

Knockdown was achieved using Mcl-1L specific siRNA (sc-43912) along with a control siRNA (sc-37007) from Santa Cruz biotechnology, USA. The siRNA duplexes were transfected using Lipofectamine-2000 (Invitrogen) according to the manufacturer’s instructions. The medium was changed after 16 hrs of transfection and 24 hrs post-transfection the cells were assessed for knockdown by western blotting. The specific silencing of Mcl-1L was confirmed in three independent experiments.

### Trypan blue exclusion assay

Cells were seeded into 24-well plates at a density of 5 × 10^4^ per well and treated with Mcl-1L siRNA and/or IR as described above. Cells were trypsinized and trypan blue staining was performed after 48 hrs of treatment. The number of viable cells were counted and compared to untreated control using a hemocytometer.

### Acquired radioresistant sublines

Radioresistant sublines were generated by irradiating AW8507 & AW13516 cells with a fractionated Ionizing radiation (FIR) strategy as described earlier [18]. During development of radioresistant cell lines, cells were collected at different doses (Control, 8, 16, 24, 32, 40 Gy) and lysates from these sublines were loaded on SDS-PAGE to determine Mcl-1L expression by western blotting.

### Statistical analysis

Statistical analysis was performed by using a Student’s *t*-test analysis. The difference between means was considered statistically significant when *P <* 0*.*05. The data is illustrated as mean ± standard deviation of three independent experiments.

## Results

### Analysis of clonogenic survival of oral cell lines

**Figure 1:**
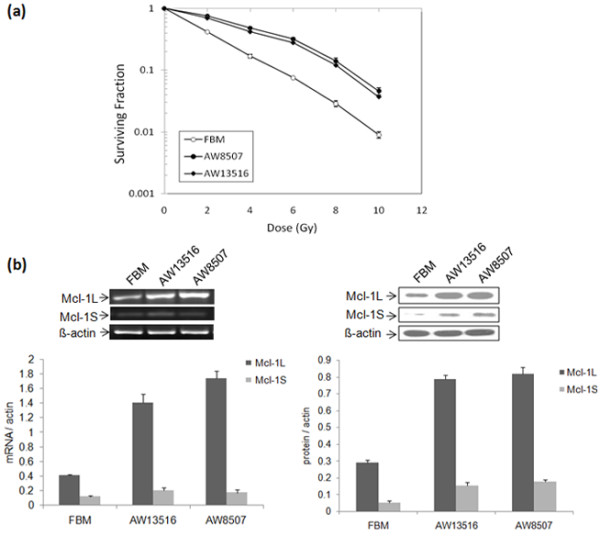
**Radiosensitivity assessment and expression of Mcl-1 isoforms in oral cell lines.** (**a**) Clonogenic cell survival assay of FBM, AW8507 & AW13516 oral cell lines. Data given as percentage survival of untreated cell cultures and represent the means (±SD) of three independent colony formation experiments. (**b**) Expression of Mcl-1 isoforms in oral cell lines. RT-PCR and western blot analysis of Mcl-1 isoforms in the three oral cell lines (FBM, AW13516 & AW8507). A representative blot was shown for three independent experiments. Histogram indicates quantitative expression of Mcl-1 isoforms (Mcl-1L & Mcl-1S) at both mRNA & protein level in oral cell lines.

Radiosensitivity assessment of the three oral cell lines (FBM, AW13516 & AW8507) used in the study was determined by the clonogenic survival assay (Figure 1a). The D_0_ values obtained from the surviving fractions of FBM, AW13516 & AW8507 were 2.3, 5.1 & 5.4 Gy respectively, indicating FBM to be most radiosensitive among all three oral cell lines.

### Expression of Mcl-1 splice variants in oral cell lines and effect of irradiation

**Figure 2:**
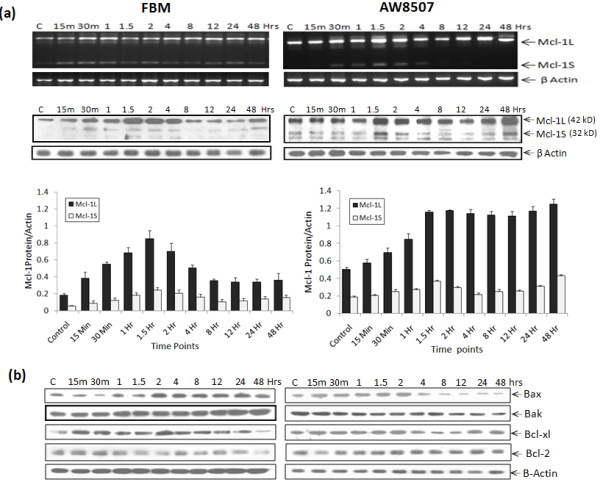
**Time course profile of Mcl-1 splice variants & apoptosis related proteins post-IR.** (**a**) Expression of Mcl-1L, Mcl-1S transcripts and proteins at different time points post-IR in FBM & AW8507. Post-IR cells were harvested at different time points, used for RT-PCR and Western blotting. (**b**) Western blot illustrates expression of Bax, Bak, Bcl-xl & Bcl-2 proteins at different time points post-IR in oral cell lines, using β-actin as loading control. A representative blot for three independent experiments is shown.

RT-PCR using a single primer which amplifies all three isoforms of Mcl-1 showed predominant expression of anti-apoptotic Mcl-1L and low levels of Mcl-1S but undetectable levels of Mcl-1ES in all three oral cell lines. Separate RT-PCR of poorly expressed Mcl-1ES isoform showed very low levels of Mcl-1ES as compared to Mcl-1L & Mcl-1S in all the three oral cell lines (Additional file 1). The more radioresistant AW8507 & AW13516 cells showed high expression of Mcl-1L at both mRNA & protein levels as compared to immortalized FBM cells (Figure 1b). Post-IR, the time course expression profiles of Mcl-1 isoforms in the three oral cell lines revealed induction of Mcl-1L protein in all three cell lines. However, radiosensitive FBM exhibited a rapid and short induction profile with a peak at 1.5 hrs which declined by 48 hrs. While the more radioresistant AW8507 exhibited sustained high levels of Mcl-1L up to 48 hrs with a peak observed at 1.5 hrs (Figure 2a). A similar pattern was observed in AW13516 (data not shown). In all cell lines, the expression of short Mcl-1S was elevated at initial time points which later decreased up to 48 hrs while, the short pro-apoptotic Mcl-1ES isoform levels remained unaltered (See Additional file 2).

### Analysis of Bax, Bcl-xl & Bcl-2 protein expression

Interestingly, AW8507 cell line exhibited a rapid downregulation of pro-apoptotic Bax & Bak proteins, 2hrs post-IR. In contrast the more radiosensitive FBM showed a consistent increase in Bax & Bak levels, 2hrs onwards (Figure 2b). Higher expression of Bcl-2 & Bcl-xl protein was observed in AW8507 as compared to FBM. The AW13516 cell line also showed similar results.

### Ratios of anti to pro-apoptotic members

**Figure 3:**
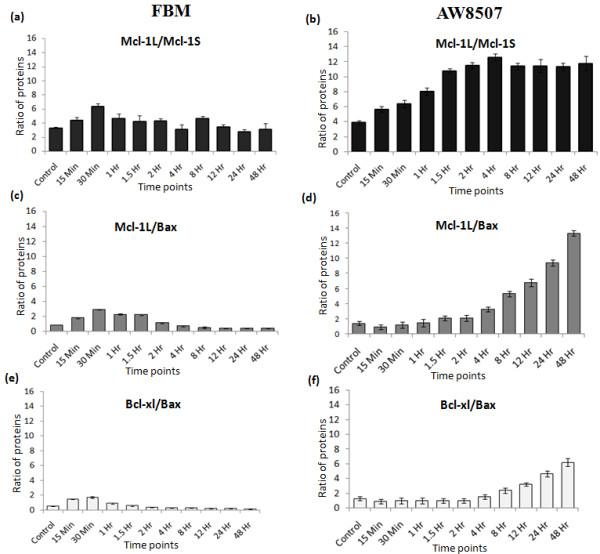
**Ratios of anti to pro-apoptotic proteins; Mcl-1L/Mcl-1S (a-b), Mcl-1L/Bax (c-d) and Bcl-xl/Bax (e-f) in AW8507 & FBM cell lines.** The relative ratios of expression of proteins were obtained by densitometry of blots using ImageJ software (NIH, USA).

It is noteworthy that, the more radioresistant AW8507 cell line exhibited higher ratios of anti to pro-apoptotic proteins like Mcl-1L/Mcl-1S, Mcl-1L/Bax & Bcl-xl/Bax as compared to that in FBM post-IR (Figure 3 a-f). A similar pattern was observed in AW13516 (data not shown).

### Effect of Mcl-1 expression on apoptosis

**Figure 4:**
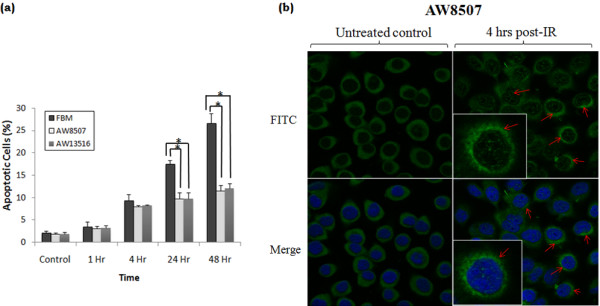
**Apoptosis induction and localization of Mcl-1 post-IR.** (**a**) Percentage of apoptosis induction at different time points post-IR by Annexin-V & PI staining analyzed by FACS. The flow cytometry data shown is representative of three independent experiments (*P < 0.05 & **P < 0.01) (**b**) Immunofluorescence staining of Mcl-1 protein counterstained with DAPI (blue) shows peri-nuclear accumulation (inset) and additional nuclear localization 4 hrs post-IR in AW8507 cells.

As compared to untreated control, post-IR up to 1 hr, a uniform time-dependent increase in apoptotic population was observed in all three oral cell lines. Notably, the number of apoptotic cells in AW13516 & AW8507 significantly (*P* < 0.05 at 24 hrs &*P* < 0.01 at 48 hrs) decreased thereafter as compared to that of FBM viz. from 17.5% to 9% at 24 hrs & 27% to 12% at 48 hrs of post-IR (Figure 4a), coinciding with the high Mcl-1L/Mcl-1S ratio (Figure 3b).

### Localization of Mcl-1 protein

Immunofluorescence staining demonstrated that Mcl-1 protein was primarily localized in the cytosolic compartment of untreated AW8507 & FBM cells. Interestingly post-IR a substantial increase in Mcl-1 protein expression with peri-nuclear and nuclear localization at 4 hrs was observed in AW8507 cells, whereas no significant change in expression and localization was observed in FBM (Figure 4b).

### siRNA mediated downregulation of Mcl-1L

**Figure 5:**
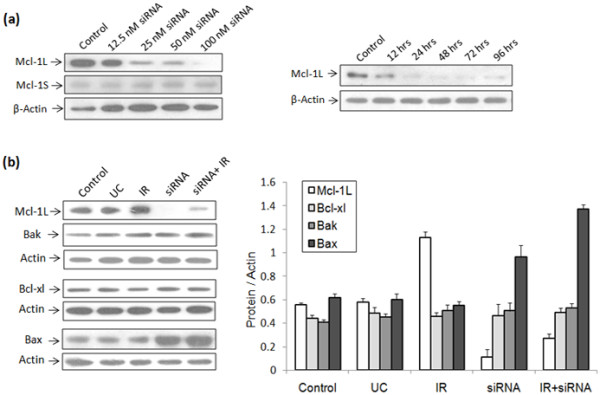
**Western blot analysis of Mcl-1L knockdown.** (**a**) Mcl-1L downregulation using different concentrations of siRNA and unaltered expression of Mcl-1S in AW8507 cells. The effect of Mcl-1L siRNA was analyzed upto 96 hrs post transfection. (**b**) Expression of Mcl-1L, Bak, Bax & Bcl-xl proteins 24 hrs after transfection of AW8507 cells: [Experimental control without siRNA (EC); universal control siRNA (UC); Mcl-1L siRNA (siRNA); irradiation (IR)]. A representative blot of three independently performed experiments is shown.

AW8507 cells exhibited specific downregulation of Mcl-1L levels after transfection with 100nM Mcl-1L siRNA without affecting the Mcl-1S levels (Figure 5a). The effect of Mcl-1L siRNA was maximal between 6 to 72 hrs and the Mcl-1S levels were unaltered. Treatment with siRNA and IR alone or in combination significantly increased expression of pro-apoptotic Bax protein but did not change Bak & Bcl-xl protein levels (Figure 5b).

### Effect Mcl-1L downregulation on cell proliferation and apoptosis

**Figure 6:**
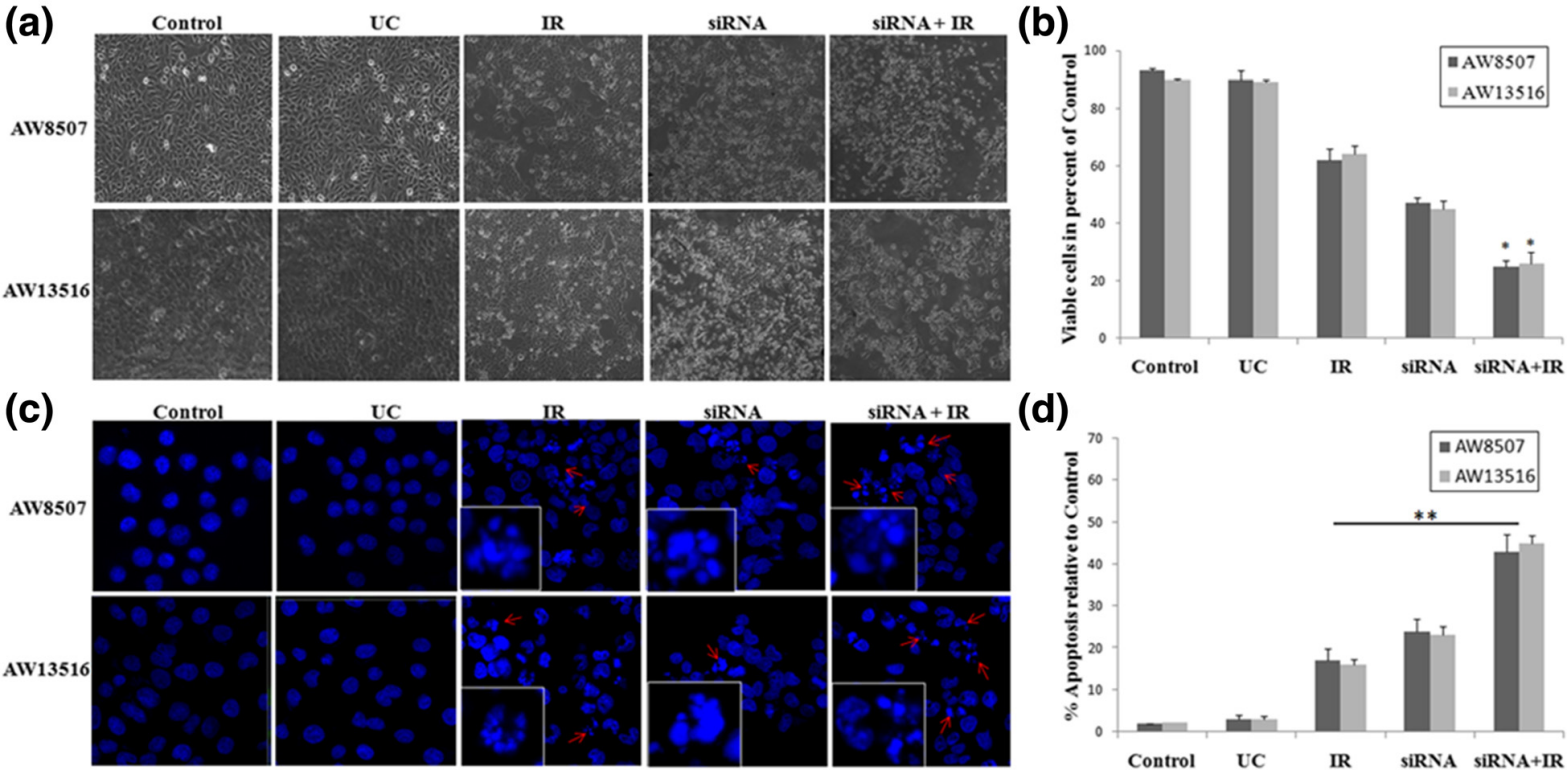
**Microscopic analyses of cell proliferation and apoptosis after different treatment combinations.** (**a & b**) After different treatment combinations as described, cell growth of AW8507 & AW13516 cells were determined by counting cell numbers using Trypan blue dye exclusion assay. Cell numbers are given in percent of untreated controls. Data are means ± SD of three independent experiments. (**c & d**) Apoptosis was determined by DAPI staining, cells either untreated (Control) or treated with UC siRNA, Mcl-1L siRNA alone or in combination with IR. Apoptotic cells were counted in different fields and shown as percent of apoptosis relative to control. Data are means ± SD of three independent experiments. *indicates *P* < 0.05, ** P < 0.01.

Trypan blue dye exclusion assay in AW13516 & AW8507 revealed a significant (*P* < 0.05) decrease in viability of cells treated with combination of siRNA plus IR as compared to individual treatments. After 72 hrs, cell viability was reduced to 67% (IR), 42% (siRNA) and 21% (IR plus siRNA) respectively (Figure 6a&b). Thereby, suggesting a synergistic effect of the combined treatment on cell viability.

Immunofluroscence analysis of AW13516 & AW8507 demonstrated an increased nuclear condensation in cells treated with combined Mcl-1L siRNA plus IR as compared to IR or siRNA alone (Figure 6c). The percentage of apoptotic cells in experimental control (EC), UC, IR, siRNA, siRNA plus IR treated AW8507 cells were 2.1% (SD 0.5), 3.2% (SD 0.6), 17.3% (SD 0.5), 25.3% (SD 1.1) and 46.3% (SD 0.6), respectively (Figure 6d). A similar pattern was observed in AW13516. The difference in percentage of apoptosis between IR alone and siRNA plus IR treated cells was highly significant (*P* < 0.01) in both the cell lines.

### Effect of Mcl1L knockdown on clonogenic survival

**Figure 7:**
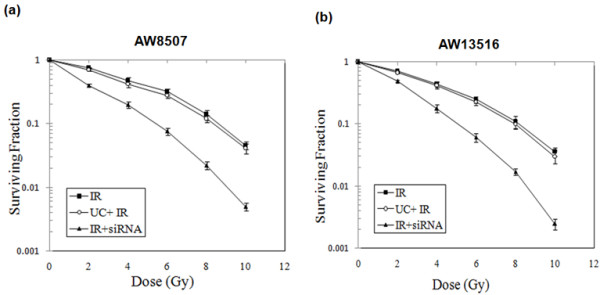
**Clonogenic survival analysis after Mcl-1L knockdown and/or irradiation.** (**a & b**) AW8507 & AW13516 cells were either untreated, or treated with siRNA. After 24 hrs, cell culture dishes were treated with increasing doses of ionizing irradiation (0, 2, 4, 6 8 and 10 Gy) as indicated. Survival was assessed by performing clonogenic assay as described in Methods. Data are given as percentage survival of untreated cells and represent the means (±SD) of three independent colony formation experiments.

The effect of Mcl-1L downregulation on long term cell survival was examined by clonogenic assay in AW8507 & AW13516 cells. Interestingly, a reduction in clonogenic survival was observed after treatment of Mcl-1L siRNA (100nM) and increasing doses of IR as compared to the untreated control (Figure 6). The survival of AW8507 post-IR (0, 2, 4, 6 8 and 10Gy) was 78% (SD 2.1), 46% (SD 1.5), 32% (SD 2.3) and 14% (SD 1.8) and 6% (SD 1.9) respectively. However, in presence of Mcl-1 siRNA the survival was further reduced to 42% (SD 2.6), 23% (SD 1.7), 10% (SD 1.0), 4% (SD 2.1) and 2% (SD 1.7), respectively (Figure 7a). Similar reduction in clonogenic survival post Mcl-1L knockdown was observed in AW13516 cells (Figure 7b). These observations therefore suggest a synergistic effect of the Mcl-1L siRNA with IR on radiosensitivity.

### Expression of Mcl-1L in radioresistant sublines

**Figure 8:**
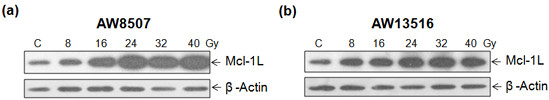
**Expression of Mcl-1L in radio resistant sublines.** (**a & b**) AW8507 & AW13516 cells were treated with fractionated irradiation and the radio resistant sublines were assayed for expression of Mcl-1L. β-actin blot from the same lysates serve as loading control.

To evaluate the association of Mcl-1L with radioresistance, its expression was assessed by western blotting in acquired radioresistant sublines of AW8507 & AW13516. Figure 8 demonstrates the high Mcl-1L expression in radio-resistant sublines generated by fractionated irradiation as compared to parental untreated cells.

## Discussion

In the present study, we demonstrate the effect of anti-apoptotic Mcl-1L expression on radiosensitivity of oral cancer cells. So far, limited information is available on the role of Mcl-1 in radiation response of tumor cells. To our knowledge, this is the first study to report a time course expression of Mcl-1 isoforms post-IR and effect of Mcl-1L knockdown on radiosentitzation of oral cancer cell lines using siRNA strategy. Our studies demonstrated an inverse correlation of Mcl-1 expression with cellular apoptosis and a synergistic effect of Mcl-1L knockdown along with IR on cell viability and clonogenic survival thereby enhancing the radiosensitivity of OSCC cells.

Various growth factors and cellular stresses like radiation and cytotoxic agents are known to upregulate Mcl-1 levels, thereby enhancing short term viability [19]. Our earlier studies had demonstrated higher expression of Mcl-1L transcript and its association with poor disease free survival in patients treated with definitive radiotherapy [10,11]. In the present study, two tongue cancer (AW8507 & AW13516) and an immortalized oral (FBM) cell line were used due to their differing radiosensitivities and based on their D_0_ values, both AW8507 & AW13516 were relatively more radioresistant than FBM. Therefore, to evaluate the association of Mcl-1L with radioresistance if any, we evaluated the expression of Mcl-1 isoforms in radioresistant AW8507 & AW13516 as compared to radiosensitive FBM. Our studies revealed higher expression of Mcl-1L at both mRNA and protein level in relatively more radioresistant AW8507 & AW13516 cell lines versus FBM, indicating a possible association of anti-apoptotic Mcl-1L splice variant with radioresistance. Several possible mechanisms can lead to the high Mcl-1 levels. Mcl-1 is known to be rapidly induced at the transcriptional level and its mRNA has a short half life [19]. Mcl-1 is also regulated at the post-transcriptional level by micro RNAs through a mir29 binding in the 3’UTR of Mcl-1 mRNA [20]. Interestingly, the expression of mir29 was also found to be decreased in malignant cholangiocytes, favoring the increased levels of Mcl-1, which indicates the possible reason for the observed high levels of Mcl-1 mRNA in our cancer cells lines and also responsible for its immediate degradation within few hours post IR.

Mcl-1, a PEST domain containing protein is also known to undergo ubiquitin dependent degradation by the 26S proteosome and possesses a short half life of one to 3 hrs is rapidly downregulated during apoptosis [19]. Notably, the BH3 domains of MULE/LASU1 E3 ligase specifically interact with the hydrophobic BH3 binding pocket of Mcl-1 and not with other anti-apoptotic Bcl-2 family members [21] and are responsible for the constitutive turnover of Mcl-1. Such ubiquitin mediated degradation of Mcl-1 has been shown to be essential for the initiation of apoptosis, following UV damage [22]. Hence, it was interesting to study the time course expression profile of Mcl-1 isoforms and other bcl-2 family members in the above cell lines post-IR. Also, Mcl-1 protein is known to be phosphorylated by GSK-3β at Ser159, located within the PEST domain, resulting in a significant decrease in the protein half-life and leading to initiation of apoptosis [23]. Such, alterations in the phosphorylation of Mcl-1 protein by GSK3 may also contribute to the elevation of Mcl-1 levels.

Moreover, it is known that the short isoform Mcl-1S only binds to Mcl-1L possibly neutralizing its anti-apoptotic function [24]. No significant alterations in levels of Mcl-1S & Mcl-1ESwere observed post IR, indicating that the predominantly overexpressed Mcl-1L isoform alone may contribute in generation of radioresistance. Mcl-1L not only binds to Mcl-1S, but is also known to heterodimerise with pro-apoptotic Bax & Bak etc. preventing the release of cytochrome-c and subsequent apoptosis [25]. We observed a downregulation of pro-apoptotic Bax & Bak proteins in AW8507 post-IR, coinciding with decreased apoptosis, while in contrast, FBM showed an increase in Bax & Bak protein levels. We observed high expression of anti-apoptotic Bcl-xl which has already been shown to be associated with radiosensitization of colon cancer cells [26]. Thus, high expression of Bcl-xl & Bcl-2, known radioresistant factors and Mcl-1L in more radioresistant AW8507 & AW13516 than FBM may indicate their possible role in radioresistance.

We assessed the ratios of Mcl-1L/Mcl-1S, Mcl-1L/Bax, Bcl-xl/Bax, wherein radioresistant AW8507& AW13516 showed high ratios as compared to that in FBM indicating predominance of anti-apoptosis which may contribute to radioresistance. We are the first to elucidate the comparative levels of Mcl-1 isoforms and their association with radiation response in oral cell lines. The high and prolonged expression of Mcl-1L observed in AW8507 & AW13516 could possibly be due to Mcl-1 protein stabilization via binding with other proteins. Another reason could be its enhanced half life due to S159/T163 phosphorylation post-IR, reported to be crucial in nicotine mediated Mcl-1 activation and chemoresistance [27].

We observed a significant reduction in apoptosis post-IR which coincided with the high levels of Mcl-1L, indicating a possible association of Mcl-1L expression with radiation response of AW8507 & AW3516 cells. The immunofluorescence staining of Mcl-1 indicated a peri-nuclear accumulation & nuclear localization post IR in more radioresistant AW8507 cell line. Such, peri-nuclear accumulation of Mcl-1 has also been observed earlier in polymorphonuclear leukocytes post-etoposide treatment [28]. In AW8507, the observed nuclear and perinuclear accumulation of Mcl-1 may possibly help in cell survival to lower doses of DNA damaging agents. A similar regulatory role for Mcl-1 (snMcl-1), perhaps acting as an adaptor protein in controlling the ATR-mediated regulation of DNA damage checkpoint kinase Chk1 phosphorylation and activation has been reported, placing Mcl-1 at the interface of apoptosis and cell cycle regulation [29]. Mcl-1 has been shown to regulate cell cycle by binding to proteins like CDK1 & PCNA [30], possibly explaining the observed nuclear localization of Mcl-1. High expression of anti-apoptotic Mcl-1L and Bcl-xl proteins and reduced pro-apoptotic proteins like Bak & Bax together may possibly contribute in lowering the sensitivity of AW8507 & AW3516 cells to IR.

The downregulation of Mcl-1L alone was efficient in induction of apoptosis in both AW8507 & AW13516 cells. Interestingly, the combination of Mcl-1L siRNA plus IR induced significantly higher apoptosis as compared to siRNA or IR-alone in both oral cell lines. Notably, the expression of closely related Bcl-xl, a known radioresistant factor was not altered. However, the expression of pro-apoptotic Bax protein correlated with the increased apoptosis on Mcl-1L knockdown. This overexpression of Bax, a downstream pro-apoptotic member, may execute the intrinsic apoptotic pathway resulting in increased cell death. To address the fact that the induction of apoptosis may not necessarily lead to long-term response to radiotherapy, we performed the clonogenic assay which demonstrated that combination of IR and Mcl-1L downregulation synergistically reduced clonogenic survival as compared to each treatment alone. Our studies demonstrate that Mcl-1L downregulation potentially enhanced radiosensitivity of AW8507 & AW13516 cells *in vitro*. Complex interactions occur between Bcl-2 family proteins especially, Bak & Bax, where Mcl-1 plays a crucial role in engaging and maintaining pro-apoptotic Bak in an inactive state and accumulates H2AX and ATM proteins to activate DNA repair pathways, suggesting that elimination of cellular Mcl-1 is crucial to initiate apoptotic pathway [31]. Overexpression and nuclear accumulation of Mcl-1 in AW8507 may occur due to a protein called IEX-1 which has been shown to interact specifically and timely with Mcl-1 controlling its accumulation and nuclear translocation in response to DNA damage and contribute in the activation of DNA repair pathway by Chk1 activation and G2 checkpoint arrest [32].

The high expression of Mcl-1L, in radioresistant sublines developed by fractionated ionizing radiation provides a direct evidence for the role of Mcl-1L in radioresistance of OSCC cells. Therefore, the combination of radiotherapy and Mcl-1L downregulation has the potential to improve the response rate of treatment-resistant oral cancer cells. Selective inhibitors like Obatoclax, which specifically overcome Mcl-1 mediated resistance, is already in phase 2 clinical trials [33] and may have important therapeutic implications, when used in combination with radiotherapy in treatment of oral cancer patients.

## Conclusion

Our studies indicate the association of Mcl-1L isoform expression with radioresistance by influencing apoptosis, proliferation and clonogenic survival of OSCC. Thus, Mcl-1L appears to be a promising molecular target for improving outcome of radiotherapy in oral cancer patients.

## Abbreviations

Post-IR: Post Irradiation; FIR: Fractionated Ionizing Radiation; IR: Ionizing Radiation; IMDM: Iscove's Modified Dulbecco's Media; FBS: Fetal bovine serum; PBS: Phosphate-buffered saline; PVDF: Polyvinyldene fluoride; DAPI: 4'-6-Diamidino-2-phenylindole; SD: Standard deviation; SDS-PAGE: Sodium dodecyle sulfate polyacrylamide gel electrophoresis.

## Competing interests

Both authors declare that they have no competing interests.

## Authors’ contributions

VP aided in study design, performed experimental procedures, analyzed the data and drafted manuscript. TT designed the study and reviewed the manuscript. All authors read and approved the final manuscript.

## Supplementary Material

Additional file 1 **Figure S1.** Indicating expression of Mcl-1ES isoform in three oral cell lines done by separate RTPCR. Click here for file

Additional file 2 **Figure S2.** Time course profile of Mcl-1ES splice variant (done by separate PCR) along with another two (Mcl-1L & Mcl-1S in single PCR) isoforms, at different time point’s post-IR in AW8507 & FBM cell lines. Click here for file

## References

[B1] Tandle AT Sanghvi V Saranath D Determination of p53 genotypes in oral cancer patients from India Br J Cancer 2001 84 739 742 10.1054/bjoc.2000.1674 11259085 PMC2363816

[B2] Burri RJ Lee NY Concurrent chemotherapy and radiotherapy for head and neck cancer Expert Rev Anticancer Ther 2009 9 293 302 10.1586/14737140.9.3.293 19275508

[B3] Mathew Iype E Pandey M Mathew A Thomas G Krishnan Nair M Squamous cell cancer of the buccal mucosa in young adults Br J Oral Maxillofac Surg 2004 42 185 189 15121260 10.1016/j.bjoms.2004.02.008

[B4] Kyprianou N King E Bradbury D Rhee J bcl-2 over-expression delays radiation-induced apoptosis without affecting the clonogenic survival of human prostate cancer cells Int J Cancer 1997 70 341 348 10.1002/(SICI)1097-0215(19970127)70:3<341::AID-IJC16>3.0.CO;2-I 9033638

[B5] Wang ZB Zhang Y Liu YQ Guo Y Xu H Dong B Cui YF Bcl-xL overexpression restricts gamma-radiation-induced apoptosis Cell Biol Int 2006 30 15 20 10.1016/j.cellbi.2005.08.006 16253528

[B6] Craig R MCL1 provides a window on the role of the BCL2 family in cell proliferation, differentiation and tumorigenesis Leukemia 2002 16 444 454 10.1038/sj.leu.2402416 11960321

[B7] Sieghart W Losert D Strommer S Cejka D Schmid K Rasoul-Rockenschaub S Bodingbauer M Crevenna R Monia BP Peck-Radosavljevic M Wacheck V Mcl-1 overexpression in hepatocellular carcinoma: a potential target for antisense therapy J Hepatol 2006 44 151 157 10.1016/j.jhep.2005.09.010 16289418

[B8] Derenne S Monia B Dean N Taylor J Rapp M Harousseau J Bataille R Amiot M Antisense strategy shows that Mcl-1 rather than Bcl-2 or Bcl-xL is an essential survival protein of human myeloma cells Blood 2002 100 194 10.1182/blood.V100.1.194 12070027

[B9] Cho-Vega JH Rassidakis GZ Admirand JH Oyarzo M Ramalingam P Paraguya A McDonnell TJ Amin HM Medeiros LJ MCL-1 expression in B-cell non-Hodgkin's lymphomas Hum Pathol 2004 35 1095 1100 10.1016/j.humpath.2004.04.018 15343511

[B10] Mallick S Patil R Gyanchandani R Pawar S Palve V Kannan S Pathak KA Choudhary M Teni TR Human oral cancers have altered expression of Bcl-2 family members and increased expression of the anti-apoptotic splice variant of Mcl-1 J Pathol 2009 217 398 407 10.1002/path.2459 19009587

[B11] Mallick S Agarwal J Kannan S Pawar S Kane S Teni T PCNA and anti-apoptotic Mcl-1 proteins predict disease-free survival in oral cancer patients treated with definitive radiotherapy Oral Oncol 2010 46 688 693 10.1016/j.oraloncology.2010.04.003 20729132

[B12] Guoan X Hanning W Kaiyun C Hao L Adenovirus-mediated siRNA targeting Mcl-1 gene increases radiosensitivity of pancreatic carcinoma cells in vitro and in vivo Surgery 2010 147 553 561 10.1016/j.surg.2009.10.033 20004446

[B13] Skvara H Thallinger C Wacheck V Monia BP Pehamberger H Jansen B Selzer E Mcl-1 blocks radiation-induced apoptosis and inhibits clonogenic cell death Anticancer Res 2005 25 2697 2703 16080514

[B14] Kim JH Sim SH Ha HJ Ko JJ Lee K Bae J MCL-1ES, a novel variant of MCL-1, associates with MCL-1L and induces mitochondrial cell death FEBS Lett 2009 583 2758 2764 10.1016/j.febslet.2009.08.006 19683529

[B15] Tatake RJ Rajaram N Damle RN Balsara B Bhisey AN Gangal SG Establishment and characterization of four new squamous cell carcinoma cell lines derived from oral tumors J Cancer Res Clin Oncol 1990 116 179 186 10.1007/BF01612674 1691185 PMC12200902

[B16] Raul U Sawant S Dange P Kalraiya R Ingle A Vaidya M Implications of cytokeratin 8/18 filament formation in stratified epithelial cells: induction of transformed phenotype Int J Cancer 2004 111 662 668 10.1002/ijc.20349 15252834

[B17] Franken N Rodermond H Stap J Haveman J van Bree C Clonogenic assay of cells in vitro Nat Protoc 2006 1 2315 2319 10.1038/nprot.2006.339 17406473

[B18] Fukuda K Sakakura C Miyagawa K Kuriu Y Kin S Nakase Y Hagiwara A Mitsufuji S Okazaki Y Hayashizaki Y Yamagishi H Differential gene expression profiles of radioresistant oesophageal cancer cell lines established by continuous fractionated irradiation Br J Cancer 2004 91 1543 1550 10.1038/sj.bjc.6602187 15365572 PMC2409931

[B19] Opferman JT Unraveling MCL-1 degradation Cell Death Differ 2006 13 1260 1262 10.1038/sj.cdd.4401978 16710358

[B20] Mott JL Kobayashi S Bronk SF Gores GJ mir-29 regulates Mcl-1 protein expression and apoptosis Oncogene 2007 26 6133 6140 10.1038/sj.onc.1210436 17404574 PMC2432524

[B21] Warr MR Acoca S Liu Z Germain M Watson M Blanchette M Wing SS Shore GC BH3-ligand regulates access of MCL-1 to its E3 ligase FEBS Lett 2005 579 5603 5608 10.1016/j.febslet.2005.09.028 16213503

[B22] Nijhawan D Fang M Traer E Zhong Q Gao W Du F Wang X Elimination of Mcl-1 is required for the initiation of apoptosis following ultraviolet irradiation Genes Dev 2003 17 1475 10.1101/gad.1093903 12783855 PMC196078

[B23] Maurer U Charvet C Wagman AS Dejardin E Green DR Glycogen synthase kinase-3 regulates mitochondrial outer membrane permeabilization and apoptosis by destabilization of MCL-1 Mol Cell 2006 21 749 760 10.1016/j.molcel.2006.02.009 16543145

[B24] Bae J Leo CP Hsu SY Hsueh AJ MCL-1S, a splicing variant of the antiapoptotic BCL-2 family member MCL-1, encodes a proapoptotic protein possessing only the BH3 domain J Biol Chem 2000 275 25255 25261 10.1074/jbc.M909826199 10837489

[B25] Thomas LW Lam C Edwards SW Mcl-1; the molecular regulation of protein function FEBS Lett 2010 584 2981 2989 10.1016/j.febslet.2010.05.061 20540941

[B26] Wacheck V Selzer E Gunsberg P Lucas T Meyer H Thallinger C Monia BP Jansen B Bcl-x(L) antisense oligonucleotides radiosensitise colon cancer cells Br J Cancer 2003 89 1352 1357 10.1038/sj.bjc.6601254 14520471 PMC2394316

[B27] Zhao J Xin M Wang T Zhang Y Deng X Nicotine enhances the antiapoptotic function of Mcl-1 through phosphorylation Mol Cancer Res 2009 7 1954 1961 10.1158/1541-7786.MCR-09-0304 19903766 PMC2796280

[B28] Leuenroth SJ Grutkoski PS Ayala A Simms HH The loss of Mcl-1 expression in human polymorphonuclear leukocytes promotes apoptosis J Leukoc Biol 2000 68 158 10914504

[B29] Jamil S Sobouti R Hojabrpour P Raj M Kast J Duronio V A proteolytic fragment of Mcl-1 exhibits nuclear localization and regulates cell growth by interaction with Cdk1 Biochem J 2005 387 659 10.1042/BJ20041596 15554878 PMC1134995

[B30] Yang-Yen HF Mcl-1: a highly regulated cell death and survival controller J Biomed Sci 2006 13 201 204 10.1007/s11373-005-9064-4 16456709

[B31] Cuconati A Mukherjee C Perez D White E DNA damage response and MCL-1 destruction initiate apoptosis in adenovirus-infected cells Genes Dev 2003 17 2922 2932 10.1101/gad.1156903 14633975 PMC289151

[B32] Pawlikowska P Leray I de Laval B Guihard S Kumar R Rosselli F Porteu F ATM-dependent expression of IEX-1 controls nuclear accumulation of Mcl-1 and the DNA damage response Cell Death Differ 2010 17 1739 1750 10.1038/cdd.2010.56 20467439 PMC3923326

[B33] Paik PK Rudin CM Pietanza MC Brown A Rizvi NA Takebe N Travis W James L Ginsberg MS Juergens R A phase II study of obatoclax mesylate, a Bcl-2 antagonist, plus topotecan in relapsed small cell lung cancer 2011 Lung cancer, Amsterdam, Netherlands 74 481 10.1016/j.lungcan.2011.05.005 PMC3715068 21620511

