# Supplementary figures and images for: Association of anti-apoptotic Mcl-1L isoform expression with radioresistance of oral squamous carcinoma cells
Source: Radiat Oncol. 2012 Aug 8;7:135. doi: 10.1186/1748-717X-7-135 (PMC3487741; doi:10.1186/1748-717X-7-135)

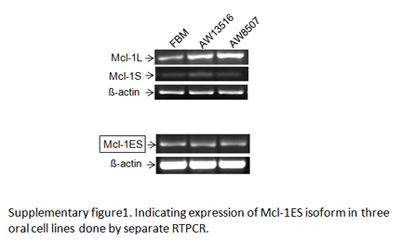

Supplement: Additional file 1 — Figure S1. Indicating expression of Mcl-1ES isoform in three oral cell lines done by separate RTPCR. [file 1748-717X-7-135-S1.tiff]

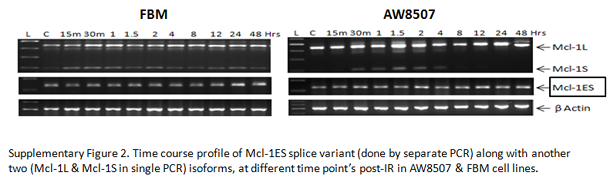

Supplement: Additional file 2 — Figure S2. Time course profile of Mcl-1ES splice variant (done by separate PCR) along with another two (Mcl-1L & Mcl-1S in single PCR) isoforms, at different time point’s post-IR in AW8507 & FBM cell lines. [file 1748-717X-7-135-S2.tiff]
